# The association of the persecutory ideation questionnaire with clinically-relevant and other outcomes: the moderating role of confidence

**DOI:** 10.1038/s41598-024-66846-9

**Published:** 2024-07-09

**Authors:** Darío Díaz, Pablo Briñol, Miriam Bajo, Maria Stavraki, Luis Beato-Fernández, Richard E. Petty

**Affiliations:** 1 Department of Psychology, Ciudad Real Medical School, Universidad de Castilla la Mancha, Camino de Moledores S/N, 13071 Ciudad Real, Spain; 2 Universidad Autónoma de Madrid, Madrid, Spain; 3 Hospital General Universitario de Ciudad Real, Universidad de Castilla la Mancha, Ciudad Real, Spain; 4 Ohio State University, Columbus, USA

**Keywords:** Psychology, Human behaviour

## Abstract

One of the most used self-administered instruments to assess persecutory delusions is the Persecutory Ideation Questionnaire (PIQ). Individual differences in PIQ scores are important because they predict the severity of symptoms associated with psychosis-related disorders. The current research demonstrates that PIQ is associated with two new outcomes: Satisfaction with life (Studies 1 and 2) and therapy length needed for hospital discharge (Study 2). Most relevant, we introduce meta-cognitive confidence in one’s scale responses as a construct capable of improving the predictive validity of the PIQ. Across two studies, participants from the general population (Study 1) and from a clinical sample (Study 2) completed the PIQ and then reported the confidence in their responses. As expected, the PIQ was associated with satisfaction with life in both cases and duration of therapy required to receive hospital discharge for the clinical sample. Most importantly, confidence further moderated the extent to which the PIQ scores were linked with both outcomes, with greater consistency between the PIQ and the dependent measures obtained for those with higher confidence. Therefore, asking a single item about the confidence associated with responses to the PIQ enhances the association of PIQ scores and relevant consequences across domains.

## Introduction

Paranoid delusional thinking is one of the core symptoms of psychosis. Delusions can occur in a wide spectrum of individuals from a non-pathological population^1^ to patients with, among others, delusional disorder, brief psychotic disorder, schizophreniform disorder, schizophrenia, schizoaffective disorder, substance/medication-induced psychotic disorder and various personality disorders (e.g., paranoid personality disorder)^2^. These delusional thoughts have traditionally been considered false beliefs (not based on rational arguments, and objective evidence), that are difficult to change, and that are often expressed with absolute conviction^3^.

Beyond expressing delusional thoughts with conviction, individuals with delusions also tend to have a cognitive style associated with feelings of confidence. In fact, this confirmation bias is a common feature in the general population, being even more pronounced in those with paranoid delusions^4,5^. For example, when processing information, people with paranoid delusions tend to pay even more attention to information that confirms their thoughts than in the general population, and have many difficulties in considering alternative viewpoints, thus generating a false sense of consensus. Indeed, having thoughts in just one dimension without contemplating any alternatives can lead people to be over-confident^6–8^. Moreover, individuals with paranoid delusions tend to use a self-focused cognitive style^3^. Thoughts that are more self-relevant have been shown to be held with more confidence than less personally relevant thoughts^9^.

Although there is a wide spectrum of paranoid themes, the most frequently observed^10,11^ and studied^12–15^ is persecution. Given the importance of persecution beliefs^3,14^, different procedures have been developed as reliable and valid ways to assess it. One of the most widely used self-administered measures of persecutory paranoid thinking is the “Persecutory Ideation Questionnaire” (PIQ^16^).

Previous research has focused on variables that are associated with PIQ scores, many of which are assumed to be antecedents^17–19^. Relatively less research exists focusing on other associations with PIQ scores that are assumed to be consequences of PIQ. The few examples available in the literature are centered on the instrument’s ability to predict the severity of related disorders symptoms^16,20,21^. Although there has been some prior studies introducing positive treatments in clinical samples with paranoid delusions^22,23^, these studies did not analyze the association between the PIQ and positive mental health (e.g., satisfaction with life). Moreover, to the best of our knowledge, there is no previous research regarding the association of the PIQ with therapy length-related outcomes.

On the one hand, life satisfaction is a relevant positive outcome because it is associated with lowered risks of all-cause and natural-cause mortality^24^ and other relevant outcomes such as lower suicide attempts^25^. Moreover, according to the Complete State Model of Health^26^ clinical evaluations and interventions should not only be focused on detecting and reducing psychopathological symptoms, but also in increasing quality of life. Satisfaction with life has been used as one of the more important indicators to examine the subjective quality of life for people experiencing serious health concerns, and probably is the most adequate and appropriate construct to do it^27^. For example, Wu and Wu^28^ found evidence that satisfaction with life is a valid measure of quality of live for patients with schizophrenia.

On the other hand, therapy length is also an important outcome to examine in this context because it is related to the cost-effectiveness associated with treatments^29^. Understanding how long therapy lasts and when a treatment has reached a good enough level is an important criterion not only for economic purposes but also for its clinical implications^30^. The dominant perspectives about how to predict therapy length are the dose–effect model^31^ and the good-enough level (GEL) model^32^ (see^33^ for a comparison between both models). The GEL model predicts that patients remain in therapy until they, in conjunction with their therapist, determine that they have improved sufficiently—to the good-enough level. Therefore, on average the amount/length of treatment reflects treatment response and indicates how malleable patients’ symptoms are, rather than being the driving force of treatment response as in the dose– effect model. Thus, the GEL model predicts that patients who respond best to treatment receive low doses of treatment, whereas patients who respond worst are the ones that need more time of treatment^30^.

As previously mentioned, paranoid delusional thinking is often linked with feelings of meta-cognitive confidence. That is, people have initial thoughts (primary cognitions) and they can subsequently think about those thoughts (secondary cognition or meta-cognition). Following a primary thought, people can also generate other thoughts that occur at a second level and play a critical role in delusional disorders^7^. Meta-cognition refers to these second order thoughts, or thoughts about one’s primary thoughts. Therefore, thought confidence is a meta-cognition because it involves thinking about the validity of initial cognition. Previous literature about thought confidence conducted with a general population suggests that mental constructs predict judgments and behaviors better when people have higher (vs. lower) confidence in their thoughts^34^. According to Self-Validation Theory (SVT)^35^, confidence can moderate the correspondence between mental contents and judgments, including, in this case, the link between traits and behavior.

Research on SVT has revealed that confidence can apply to any mental construct. This work has shown that mental contents are especially predictive of judgment and behavior the greater the perceived validity that individuals have in their thoughts. These perceptions of validity can be assessed easily by asking people to rate the confidence or certainty they have in their responses to any inventory. Perceptions of validity have been useful in moderating the effects of individual differences in different domains (e.g., political ideology^36^, group processes^37^, health behaviors^38^). As an illustration of this body of research guided by the SVT framework, some research^38^ has shown that responses of participants within the general population to a porn scale were associated with porn consumption to a greater extent for people with high (vs. low) confidence in their responses to the scale. In another illustration^39^, research has shown that participants’ trait aggressiveness in a general population sample was more predictive of actual aggressive behavior as individuals’ confidence in their own responses to the aggressiveness inventory increased. The logic behind these findings is that people think about their traits along with the associated confidence when deciding how to act. In other words, mental contents are especially predictive of behavior the greater the perceived validity that individuals have in their thoughts. For example, people might think how much porn they like to consume or how aggressive they are before deciding to act and to the extent that they have confidence in that thought about how they are, they will act more in accord with it when the relevant situation comes.

To address the role of confidence in understanding PIQ outcomes, the current work had two core goals. The first is to analyze the relationship between the PIQ and satisfaction with life, and the link between the PIQ and therapy length to obtain hospital discharge. Second, an important goal is to test whether confidence in responses to the PIQ can moderate these relationships in the same way that confidence has moderated the impact of other individual differences on postulated outcomes. Study 1 examined to what extent confidence linked to the responses given to the PIQ can help to explain the conditions under which the relationship between the scale and satisfaction with life will be stronger. Study 2 tested this question in a clinical sample diagnosed with persecution delusions. Also, Study 2 included a behavioral measure related to paranoid delusions, the duration of therapy required to receive hospital discharge.

## STUDY 1

The goals of Study 1 were to test the extent to which PIQ served to detect positive mental health in the general population, and to examine whether confidence in scale responses could strengthen the predictive validity of the inventory. First, participants answered the PIQ, and then reported the extent to which they were confident about their responses to the instrument (meta-cognitive confidence). These two variables and their interaction served as predictors of satisfaction with life. Consistent with SVT, we expected that as participants’ confidence in PIQ answers increased, responses to the instrument would be more associate with life-satisfaction.

## Methods

### Participants

Two hundred people from the Spanish general population (132 females, 67 males, 1 unidentified) participated voluntarily, anonymously and without any compensation (*M*_*age*_ = 46.81, *SD* = 6.04). Participants were recruited through letters of invitation explaining the project and the voluntary nature of the participation. The invitations were given by undergraduate medical students (Universidad de Castilla La Mancha) to their family members. A power analyses was conducted using G*Power. Because no prior research had examined the interaction between the PIQ and confidence we planned for a generic medium effect (*f *^2^ = 0.04). The results of the power analysis concluded that the desired sample size for a two-tailed test (*α* = 0.05) of the predicted two-way interaction with 0.80 power was N = 199 participants. In the end, we exceeded this recommendation slightly by obtaining two hundred participants.

### Procedure

Participants received information about the purpose of the study explaining that the goal of the research was to validate a standardized test. Participants first completed the Persecutory Ideation Questionnaire^16^. Then, participants reported their confidence in their responses to the scale (see *Predictor Variables* for a complete description of this variable), after which they completed the Satisfaction with Life Scale^40^. Finally, participants were debriefed, thanked, and dismissed.

### Predictor variables

#### Persecutory ideation questionnaire (PIQ)

We used the Spanish version of the “Persecutory Ideation Questionnaire” (PIQ)^16^ previously validated by Fonseca-Pedrero and colleagues^41^. The PIQ is a 10-item instrument that includes statements about paranoid thinking such as: “People mean to do and say things to annoy me” or “Some people try to steal my ideas and take credit for them.” The original PIQ and the Spanish version have been evaluated in previous studies^16,41^ using clinical and general population samples, showing in both cases adequate internal consistency, unidimensional factorial validity, and criterion validity as a measure of paranoid style focused on persecutory ideation. Participants answered using a 4-point scale (0 = completely false to 3 = completely true). In our study we computed the mean of the 10 items of the PIQ, therefore the maximum possible score is 3, and the minimum score is 0. Higher scores indicate more persecutory ideation. The mean of the PIQ in Study 1 (*M*_*PIC*_) was 0.99 (*SD* = 0.65). This value was equivalent to the mean values observed for this scale in other studies conducted with general population samples. For example, in the original study of the PIQ, the mean value of the general sample was 0.91 (range 0–4) (*SD* = 0.60)^16^. Subsequent studies have obtained similar mean values (e.g., Fernyhough et al.^42^: *M*_*PIC*_ = 1.01, *SD* = not reported; Jones & Fernyhough^43^: *M*_*PIC*_ = 0.87, *SD* = 0.68; Jones et al.^17^: e-questionnaire *M*_*PIC*_ = 0.72, *SD* = 0.51, pen and pencil questionnaire *M*_*PIC*_ = 0.82, *SD* = 0.63 or Van Dongen et al.^44^: *M*_*PIC*_ = 0.33, *SD* = 0.33). In the present study, Cronbach’s *α* value for the PIC scale was 0.93. In a median split, for people with low confidence scores Cronbach’s *α* value was 0.92 and for people with high confidence scores it was 0.93.

#### Confidence

Participants indicated the confidence they had in their responses to the PIQ by answering the following item: “How confident are you in the responses you just gave to the scale?” (1 = “Extremely unconfident” to 9 = “Extremely confident”). Thus, higher scores on this item indicate greater confidence (*M*_*TC*_ = 6.33, *SD* = 1.99). This measure of confidence was identical to the one used by in prior research^36–39^. In previous SVT research conducted with a general population, confidence was measured with this question, and this item was found to moderate the predictive validity of other inventories relevant to group behavior and to moderate the association of other mental constructs unrelated to the present domain.

### Dependent variable

#### Satisfaction with life

The Spanish adaptation of the Satisfaction with Life Scale (SWLS)^40^ was used in this study. The scale has 5-item that includes statements about life satisfaction such as: “In most ways my life is close to my ideal” or “The conditions of my life are excellent.” Both the original SWLS scale and the Spanish version of this instrument have shown good psychometric properties in previous studies^40,45^ in terms of reliability and unidimensional factorial validity. Participants answered using a 5-point scale (1 = strongly disagree to 5 = strongly agree) with higher values indicating more favorable evaluations of life. In the present study we computed the mean of the 5 items of the scale. Therefore, the maximum possible score is 5 and the minimum is 1, with higher indicating scores more life satisfaction. Cronbach’s *α* value for the SWLS scale was 0.91 (*M*_*WB*_ = 3.35, *SD* = 0.98).

### Ethics approval

The study was approved by the ethics committee of the “Universidad de Castilla - La Mancha” (UCLM) and the Hospital General Universitario de Ciudad Real (“Comité Ético de Investigación Clínica HGUCR-UCLM”, No. 01/2020/C-305). It was performed in accordance with the ethical standards as laid down in the 1964 Declaration of Helsinki and its later amendments or comparable ethical standards.

## Results

The data for skewness of all the variables included in Study 1 ranged from 0.08 to − 0.73, and Kurtosis ranged from 0.26 to − 1.07. Therefore, according to recommended criteria^46^ (maximums of 2 for skewness and 7 for kurtosis), the variables in our studies follow a normal distribution. First, we conducted a preliminary analysis to examine the relationships among the three variables included in the study using Pearson correlations. A significant negative correlation was observed between the PIQ and satisfaction with life, *r*(198) = −0.17, *p* = 0.01. Moreover, the correlation between confidence and satisfaction with life was positive and significant, *r*(198) = 0.18, *p* = 0.01. Finally, the correlation between the PIQ and confidence was also positive and significant, *r*(198) = 0.17, *p* = 0.02.

Satisfaction with life was then subjected to a hierarchical regression analysis using the PROCESS add-on for SPSS (model 1)^47^. PROCESS is a computational procedure for SPSS and SAS that implements moderation or mediation analysis as well as their combination in an integrated conditional process model. We introduced the predictor variables (reported PIQ and confidence) at the first step, then added a computed interaction term at the second step. Since the predictors were continuous variables (i.e., PIQ and confidence) they were mean-centered prior to analyses. Finally, age and sex were included as covariates. As expected, results of the regression analysis showed a significant main effect of the PIQ on satisfaction with life, *B* = −0.35, *t*(194) = −4.09, *p* = 0.001, 95% CI −0.51, −0.18, indicating that as the PIQ increased, satisfaction with life decreased. The main effect of confidence on satisfaction with life was also significant, *B* = 0.21, *t*(194) = 3.17, *p* = 0.002, 95% CI 0.08, 0.35, revealing a positive association between confidence and satisfaction with life.

**Figure 1:**
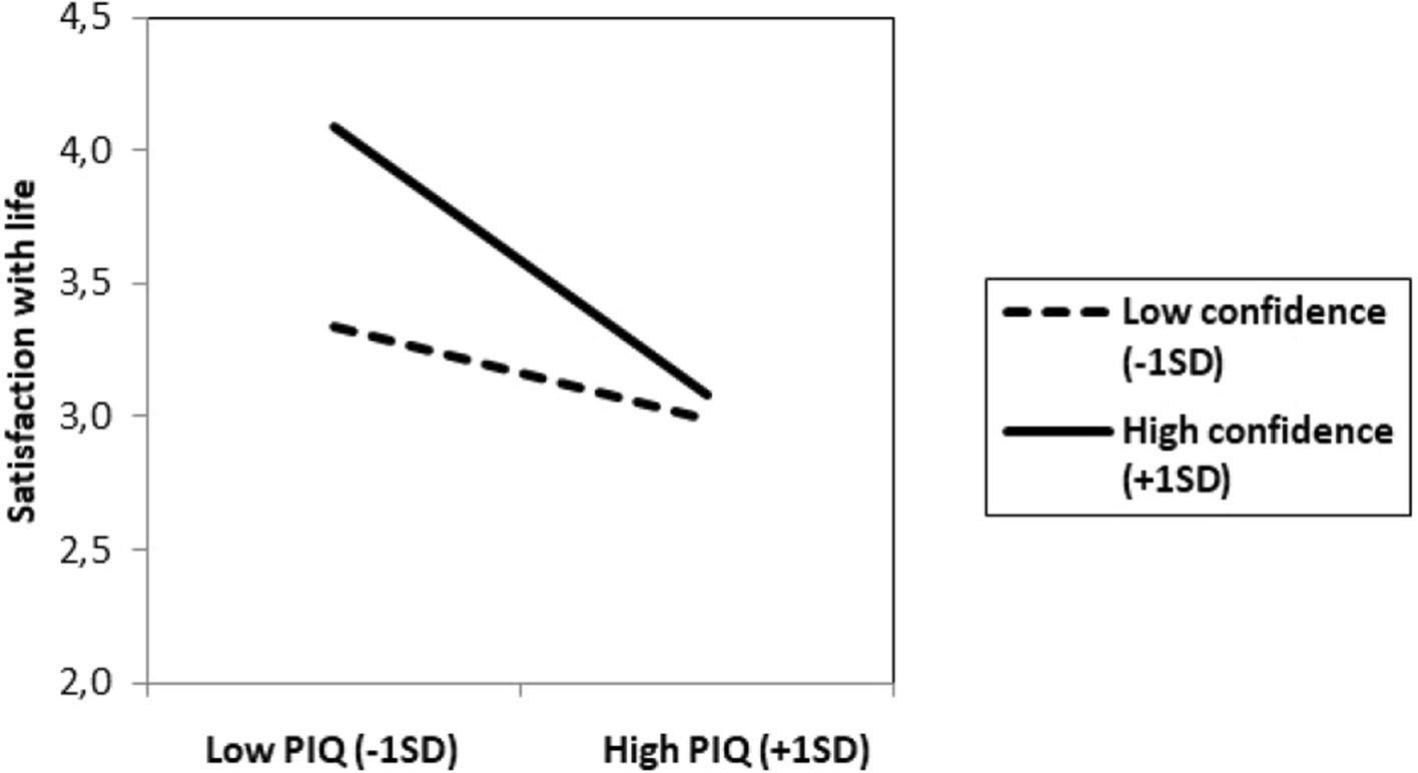
Study 1. Satisfaction with life as a function of the Persecutory Ideation Questionnaire (PIQ) and confidence.

Importantly, the predicted interaction between the PIQ and confidence was significant, *B* = − 0.17, *t*(194) = − 2.67, *p* = 0.008, 95% CI − 0.30, − 0.05. As shown in Fig. 1, among those with higher confidence (+ 1SD), the PIQ was negatively associated with satisfaction with life, *B* = − 0.52, *t*(194) =  − 5.54, *p* < 0.001, 95% CI − 0.70, − 0.33. However, for those with lower confidence scores (− 1SD), the expected relationship between the PIQ and satisfaction with life was no longer significant, *B* = − 0.18, *t*(194) =  − 1.50, *p* = 0.13, 95% CI − 0.41, 0.05.Analyzed differently, this interaction showed that among participants with low levels of the PIQ (− 1SD), confidence was positively related to satisfaction with life, *B* = 0.38, *t*(194) = 4.69, *p* < 0.001, 95% CI 0.22, 0.54. In contrast, for participants with high levels of the PIQ (+ 1SD), the relation between confidence and satisfaction with life was not significant, *B* = 0.04, *t*(194) = 0.41, *p* = 0.68, 95% CI − 0.16, 0.24 (for a multiple regression analysis on confidence with the PIQ, satisfaction with life and the interaction terms as predictor variables see Supplementary Material; for a multiple regression analysis on the PIQ with confidence, satisfaction with life and the interaction terms as predictor variables see Supplementary Material).

## Discussion

The results of Study 1 provided evidence of the predicted link between PIQ and satisfaction with life. Scoring high in PIQ was associated with lower life satisfaction. As noted, this outcome is important because it has been associated with a wide range of psychological and health outcomes^24,25^.

More uniquely, the effect of the PIQ on satisfaction with life measure was further moderated by confidence. As predicted, scores on persecutory delusions were negatively associated with satisfaction with life to a higher extent as participants reported higher levels of confidence in their responses to the scale. This result suggests that researchers interested in analyzing the effect of paranoid persecutory delusions on wellbeing could benefit from considering confidence. This result is also convergent with previous research that proposed rigid confidence as a harmful characteristic of paranoid thought in general population^48^.

Finally, it is worth noting that although the PIQ scores informed by participants of this first study conducted in general population were relatively low in absolute terms, the critical question is to what extent PIQ scores will be significantly lower in a general population sample compared to a clinical sample with persecutory delusions. In the next study we will employ a clinical population to analyze the capability of the PIQ to discriminate between both samples.

## STUDY 2

The first goal of Study 2 was to test the generalizability of the obtained results from a general population to a clinical sample. The second goal of this study was to test, for the first time, the possible association of the PIQ with an observed behavior, the length of the therapy required to be discharged. The third goal was examining the extent to which confidence could improve the link between PIQ and the new outcome. Again, we expected a negative relationship between PIQ and satisfaction with life, and that this relationship would be stronger as confidence increases. Regarding therapy length, we hypothesized that the PIQ would be positively related with the amount of time required to be discharged, and this relationship would also be moderated by confidence such that the impact of PIQ on discharge time would be greater as confidence increased.

## Methods

### Participants

Sixty people (25 females, 35 males; *M*_*age*_ = 39.43, *SD* = 13.40) with a SCID-5 confirmed DSM-5 diagnosis of Schizophrenia (N = 23), Brief Psychotic Disorder (N = 19), Delusional Disorder (N = 13) or Substance/Medication-Induced.

Psychotic Disorder (N = 5) were recruited in the “Hospital General Universitario de Ciudad Real” (HGUCR). All participants presented persecutory paranoid delusions. Participants’ age ranged from 18 to 76 (*M*_*age*_ = 39.95, *SD* = 13.45). Patients were hospitalized and were in a post-acute or stable phase of their disorders. Given the difficulty of accessing participants with persecutory delusions willing to sign the informed consent, we proposed to collect the maximum number of patients possible during a time interval of 18 months. This number of participants allowed us to detect a two-way interaction effect size of *f *^2^ = 0.13 with 0.8 power.

### Procedure

All candidates received a letter describing the research project based on developing inventories and the voluntary nature of participation. Also, they were informed that the decision to participate (or not) in the study would not influence the clinical assistance received. The study was approved by the ethics committee of the “Universidad de Castilla—La Mancha” (UCLM) and the HGUCR (*Comité Ético de Investigación Clínica HGUCR-UCLM*). Those who agreed to participate were informed that all collected information in the study was confidential and anonymous. As soon as patients were stabilized and were in conditions to respond to questions, they were asked for their permission to participate in the study. Participants who volunteered to participate completed the information consent, answered the PIQ, reported their confidence in their responses to this instrument, and answered the Life Satisfaction Scale, in that order, as soon as possible within the first 48 hours after admission. The metacognitive therapy began just after the completion of the tests, and therefore the treatment could not influence the previously reported responses to the PIQ or confidence questions.

### Predictor variables

#### Persecutory ideation questionnaire (PIQ)

Persecutory delusions were measured using the same scale^16^ as in Study 1. In the present study, Cronbach’s *α* value for the PIC scale was 0.94 (*M*_*PIC*_ = 1.23, *SD* = 0.88). The mean score on the PIQ in Study 2 was within the same range of mean values observed in previous studies conducted with clinical samples. For example, in the original study of the PIQ the mean value for the clinical sample was 1.34 (range 0–4) (*SD* = 0.91)^16^. Other studies found similar results (e.g., Dudley et. al.^49^ Time 1: *M*_*PIC*_ = 1.38, *SD* = 1.00, Time 2: *M*_*PIC*_ = 1.33, *SD* = 1.01; Van Dongen et. al.^44^: *M*_*PIC*_ = 1.37, *SD* = 1.08; Van Dongen et al.^19^: *M*_*PIC*_ = 1.48, *SD* = 1.18). In a median split, for people with low confidence scores Cronbach’s *α* value was 0.95 and for people with high confidence scores was 0.93.

#### Confidence

Participants indicated their confidence using the same item as in Study 1 (*M*_*TC*_ = 6.97, *SD* = 1.97), also employed in past research^37–39^.

### Dependent variables

#### Satisfaction with life

We measured participants’ life satisfaction using the same scale as in Study 1 (SWLS)^40,45^ (*M*_*WB*_ = 2.95, *SD* = 0.92). In the present study, Cronbach’s *α* value for the SWLS scale was 0.81.

#### Therapy length required to be discharged

During hospitalization, all patients received psychological therapy based on the Well’s metacognitive model^50^. Specifically, the adaptation of this therapy for patients with schizophrenia proposed by Morrison and colleagues^51^ was used. As proposed by these authors, the duration and number of sessions varied based on the individual case formulation and as a function of improvement^51^. Therefore, longer periods of time receiving treatment before discharge indicated slower recovery and a release delay. The total length of therapy for each patient was registered in minutes. On average, patients received 561 min of metacognitive therapy before discharge (*SD* = 54 min).

## Results

The data for skewness of all the variables included in study 2 ranged from 0.52 to − 0.58, and Kurtosis ranged from − 0.19 to − 0.96. Therefore, according to recommended criteria^46^ (maximums of 2 for skewness and 7 for kurtosis), the variables in our studies follow a normal distribution. A preliminary analysis of the relationships between the variables was conducted using Pearson correlations. A negative correlation was observed between the PIQ and satisfaction with life, *r*(59) = − 0.45, *p* < 0.001, and a positive correlation between the PIQ and Therapy Length, *r*(59) = − 0.63, *p* < 0.001. Moreover, the correlations between confidence and satisfaction with life, *r*(59) = 0.30, *p* = 0.02, and between confidence and Therapy Length, *r*(59) = 0.31, *p* = 0.02, were significant. Finally, the correlation between the PIQ and confidence was also significant, *r*(59) = 0.26, *p* = 0.04.

Next, the two dependent variables were submitted to a hierarchical regression analysis using the PROCESS add-on for SPSS (model 3) following the same procedure as in Study 1, including PIQ, confidence and DSM-5 diagnosis and the interaction terms as predictor variables. The three-way interactions for satisfaction with life and therapy length were not significant, *B* = 0.01, *t*(52) = 0.15, *p* = 0.88, 95% *CI* − 0.19, 0.22; *B* = − 0.17, *t*(52) = − 1.87, *p* = 0.07, 95% *CI* − 0.36, 0.01. Therefore, the key two-ways interactions of PIQ and confidence were not moderated by DSM-5 diagnosis. For this reason, in the next analyses we introduced only the PIQ and confidence as predictor variables.

### Satisfaction with life

This dependent variable was submitted to a hierarchical regression analysis using the PROCESS add-on for SPSS (model 1) following the same procedure as in Study 1. Replicating the previous study, the main effect of the PIQ on satisfaction with life was significant, *B* = − 0.54, *t*(54) = − 5.16, *p* < 0.001, 95% CI − 0.76, − 0.33, indicating that participants scoring higher in the PIQ reported less satisfaction with life. Also, we found a main effect of confidence on satisfaction with life, *B* = 0.39, *t*(54) = 3.52, *p* < 0.001, 95% CI 0.17, 0.61, showing that higher levels of confidence correspond to higher levels of satisfaction with life.

**Figure 2:**
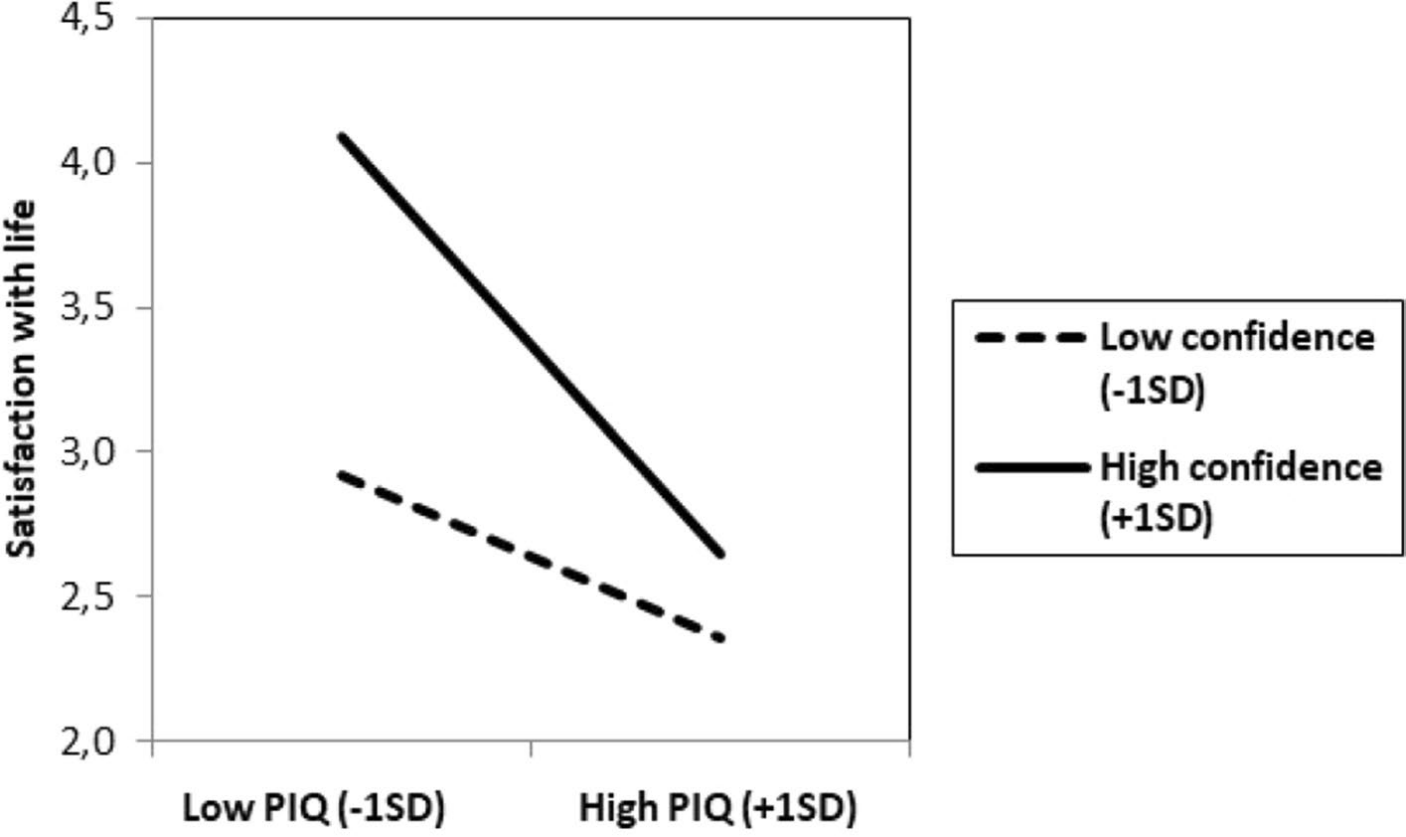
Study 2. Satisfaction with life as a function of Persecutory Ideation Questionnaire (PIQ) and confidence.

More importantly, the predicted interaction between the PIQ and confidence was significant *B* = − 0.24, *t*(56) = 2.09, *p* = 0.04, 95% CI − 0.46, − 0.01. As illustrated in Fig. 2, among those with higher confidence scores (+ 1SD), the PIQ was negatively associated with reported satisfaction with life, *B* = − 0.78, *t*(54) =  − 5.28, *p* < 0.001, 95% CI − 1.00, − 0.48. For those with lower confidence scores (− 1SD), a weak non-significant relationship emerged between the PIQ and satisfaction with life *B* = − 0.31, *t*(54) = − 1.92, *p* = 0.06, 95% CI − 0.63, 0.01.

Analyzed differently, this interaction showed that, among participants with low levels of the PIQ (− 1SD), confidence was positively related to satisfaction with life, *B* = 0.62, *t*(54) = 4.39, *p* < 0.001, 95% CI 0.34, 0.90. In contrast, for participants with high levels of the PIQ (+ 1SD), the positive relation between confidence and satisfaction with life did not emerge, *B* = 0.15, *t*(54) = 0.88, *p* = 0.39, 95% CI − 0.19, 0.49 (for a multiple regression analysis on confidence with the PIQ, satisfaction with life and the interaction terms as predictor variables see Supplementary Material; for a multiple regression analysis on the PIQ with confidence, satisfaction with life and the interaction terms as predictor variables see Supplementary Material) (See also Supplementary Material for additional analysis with the aggregated data of Studies 1 and 2).

#### Therapy length required to be discharged

Similar statistical procedures were used as in the prior regression analysis. This regression analysis revealed a main effect of the PIQ, *B* = 0.57, *t*(54) = 5.63, *p* < 0.001, 95% CI 0.37, 0.78, indicating that it was positively related to Therapy length. Also, we found a main effect of confidence, *B* = 0.20, *t*(54) = 1.96, *p* = 0.05, 95% CI 0.00, 0.41, indicating that greater confidence was associated with slower recovery and release delay (i.e., more Therapy length). Although speculative, according to the GEL Model this can be interpreted as a case in which higher reports of confidence indicates more resistance to change and a poor response to psychological treatment^30,32^.

**Figure 3:**
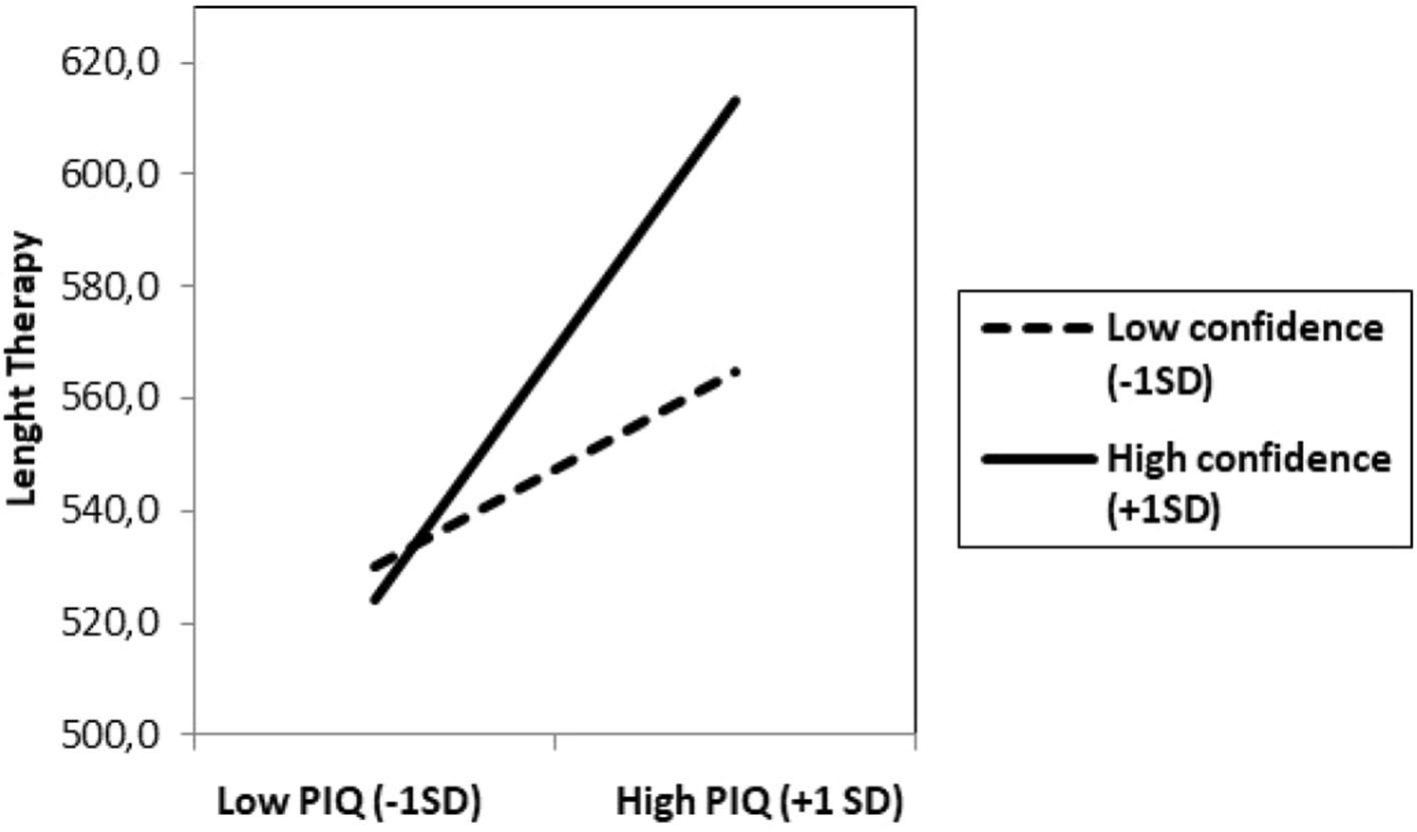
Study 2. Therapy length required to be discharged as a function of Persecutory Ideation Questionnaire (PIQ) and confidence.

More importantly, the predicted interaction between the PIQ and confidence was significant,* B* = 0.24, *t*(54) = 2.20, *p* = 0.03, 95% CI 0.02, 0.46. As illustrated in Fig. 3, for participants scoring higher in confidence (+ 1SD), the PIQ was positively associated with Therapy length, *B* = 0.81, *t*(54) = 5.71, *p* < 0.001, 95% CI 0.53, 1.00. For those scoring lower in confidence (− 1SD), there was a weaker but significant relationship between the PIQ and Therapy length,* B* = 0.35, *t*(54) = 2.15, *p* = 0.04, 95% CI 0.02, 0.65. Therefore, having higher confidence in the PIQ answers was associated with PIQ better predicting slower recovery and more need for further therapy than having lower confidence.

Analyzed differently, this interaction showed that among participants with higher levels in the PIQ (+ 1SD), higher confidence was associated with increased Therapy length, *B* = 0.44, *t*(54) = 2.66, *p* = 0.01, 95% CI 0.11, 0.77. In contrast, for participants with lower levels in the PIQ (− 1SD), no association was found between the variables, *B* = − 0.04, *t*(54) =  − 0.28, *p* = 0.78, 95% CI − 0.31, 0.24.

## Discussion

Patients’ scores on the PIQ were overall negatively associated with their satisfaction with life. This finding conceptually replicated the results observed in the first study for the general population sample and provides convergent evidence of the predictive validity of the PIQ across different populations. Most relevant to the present concerns, the association between responses on the PIQ and life satisfaction was greater as reported confidence increased. Therefore, we successfully replicated the moderation effect from Study 1 and extended it to a clinical population.

Beyond replicating results for satisfaction with life, this second study further extended the predictive validity of PIQ to other relevant outcomes. As predicted, higher scores on the PIQ were related to greater therapy length before being discharged. These results speak to the clinical relevance of this instrument. Furthermore, consistent with SVT, confidence in the responses to the PIQ moderated this relationship. That is, the higher the confidence levels in the PIQ, the stronger the relationship between the PIQ and the duration of therapy.

Finally, participants in the clinical sample reported relatively low levels of persecutory ideation, although the mean value was within the same range observed in previous studies conducted with hospitalized patients^19,44^. One possible explanation for the relatively low values in the PIQ (in absolute terms) is that clinical participants were in a post-acute or relatively stable phase of their disorders. More critically for the present research is that the mean values of the PIQ were significantly different for the clinical and general population samples, supporting the ability of this instrument to discriminate between those two types of populations.

## General discussion

Across two studies the results supported our hypothesis that the PIQ is a valid measure to predict relevant new outcomes across different types of samples. Therefore, the current research supports the predictive validity of the PIQ^16^ by showing the instrument’s ability to be negatively associated with a measure of positive mental health (i.e., life satisfaction) and clinically relevant behaviors (i.e., amount of therapy time required before discharge). Furthermore, confidence moderated the effects of informed persecutory paranoid delusions on satisfaction with life, both for general and clinical populations, and therapy length required to be discharged for a clinical population. For a general population sample (Study 1), we predicted and found that informed persecutory delusions were inversely related to satisfaction with life to a greater extent as confidence increased. Study 2 replicated this result and extended it to patients diagnosed with disorders linked to persecutory paranoid delusions. Among other implications, this is important because it is the first time that the SVT framework has been applied to clinical settings and samples.

Importantly, the PIQ, as a self-report instrument, has some limitations related, for example, to being susceptible to individual differences in self-awareness^52^ or to so-called response factors (e.g., correction in judgments^53^). For this reason, in Study 2 we also examined the instrument’s ability to predict a relatively more objective outcome (i.e., amount of therapy time required before discharge). Previous research on the PIQ mainly focused on other factors associated with the scores, and the ability of the instrument to detect symptoms severity, but there are no prior studies analyzing the measure’s capacity to predict outcomes relevant to the treatment. Of course, besides its well-established importance^33^, therapy length, like any other objective measure, has its own potential limitations of interpretation. For example, although the GEL model predicts that small doses of treatment are related to relatively fast rates of change, it could be that some patients are relieved earlier because they respond aggressively to the therapist^54^. In those possible cases, shorter times should not be interpreted as a positive outcome. Given that any measure is open to interpretation, future studies could benefit from using multiple assessments of both an objective and subjective nature to reduce the potential room for interpretation associated with each of them.

Considering that the PIQ was associated both with positive health and therapy length, our results are not only relevant to the general public but also especially relevant for clinical researchers. Moreover, in an applied context, adding a single item to the PIQ to measure confidence in responses enhances the predictive capacity of the instrument. Also, this confidence measure is easy for patients to answer^36,55^ and does not significantly increase the time needed to complete the questionnaire. It thus provides a simple and efficient way to separate metacognitive confidence from first-order delusional thoughts (i.e., responses to the PIQ inventory).

In view of our results, future research could explore the extent to which higher confidence (including pathological confidence in clinical populations, e.g., I am confident that people are plotting against me so I must use my thoughts more) coming right after the PIQ is causing participants to report less satisfaction with life or to need more therapy time before discharge, or whether the questionnaire is predicting more or less accurately when meta-cognitive confidence is high (vs. low) (e.g., higher confidence increases precision in accessing mental content related to delusions). Future studies could also examine the extent to which confidence can moderate the predictive validity of other inventories typically associated with pathological doubt, such as the Obsessive–Compulsive Inventory-Revised (OCI-R)^56^ or the Clark-Beck Obsessive Compulsive Inventory (CBOCI)^57^.

In addition to the limitations mentioned for the PIQ itself, there are other limitations to consider despite the overall importance of these results. First, the designs of both studies were correlational and there could therefore be alternative interpretations based on the involvement of third variables. It could be argued that instead of confidence increasing the relation between persecutory delusional thoughts and therapy length, a third (unmeasured) variable is related to both confidence and therapy length and is responsible for the observed relationships. Therefore, future research should manipulate confidence^31^ in addition of using a measurement approach. Nonetheless, the practical applications of measuring confidence to enhance predictive ability remain. Second, the sample size of the clinical population (Study 2) could limit the generalizability of the results. Although a wide time frame was planned for data collection, the final sample size was relatively small. This is because participants’ recruitment was difficult since patients with persecutory delusions are usually reluctant to sign an informed consent. In fact, previous studies with patients who suffer persecution delusions and schizophrenia tend to have similar or even smaller sample sizes^58^. To examine the statistical power of Study 2, we conducted a *post-hoc* analysis using G*Power. The results indicated that Study 2 (*N* = 60; Cohen’s *f*^2^ = 0.05) showed low statistical power (0.42). Thus, it is important to replicate this study.

Finally, it is important to mention that this research assumes that confidence is linked to high perceived validity. Therefore, we expect that the results obtained in these studies will be replicated when confidence is associated with high validity (e.g., being right), an association that tends to occur in paranoid delusions^3^. However, if people with paranoid delusions linked confidence to low validity (e.g., patients were aware of their mental rigidity), a different pattern of results might be obtained. Thus, the meaning of confidence is proposed to moderate the impact that confidence in one’s personality has on subsequent behavior^59^. In fact, changing the meaning of confidence is a target of different metacognitive therapies for paranoid delusions (Metacognitive Training)^60^.

Keeping meaning constant, another avenue for future research has to do with specifying when confidence validates and when doubt could potentially validate. For example, previous research has documented a possible connection between low levels of confidence and extreme compensatory behaviors^61^. In this sense, if paranoid delusions had a compensatory nature, one would expect that relatively low (vs. high) levels of confidence would be linked with more predictive power of the PIQ. However, the nature of paranoia justifies our findings^48^, that is, a high (rather than low) confidence in the PIQ enhances prediction of behavior.

To conclude, our results indicate that measuring meta-cognitive confidence can increase the association between the PIQ’s and satisfaction with life and therapy length both in clinical and general population contexts.

### Supplementary Information

Supplementary Information.

## Data Availability

The datasets generated during and/or analyzed during the current study are available from the corresponding author on reasonable request.
