# Supplementary material for: The association of the persecutory ideation questionnaire with clinically-relevant and other outcomes: the moderating role of confidence
Source: Sci Rep. 2024 Jul 9;14:15809. doi: 10.1038/s41598-024-66846-9 (PMC11233644; doi:10.1038/s41598-024-66846-9)
Supplement: Supplementary file 1 — Supplementary Information. [file 41598_2024_66846_MOESM1_ESM.docx]

Supplementary Material.

1.- *Hierarchical regression analysis on confidence with the PIQ, satisfaction with life and the interaction terms as predictor variables (Study 1).* The main effect of the PIQ on confidence was significant, *B* = .26, *t*(194) = 2.88, *p* = .005, 95% *CI*: .08, 44, indicating that participants scoring higher in the PIQ reported more confidence. Also, the main effect of satisfaction with life on confidence was significant, too, *B* = .29, *t*(194) = 4.11, *p* < .001, 95% *CI*: .15, 43, showing that participants with higher punctuations in satisfaction with life informed more confidence. The interaction between the PIQ and satisfaction with life was significant *B* = -.21, *t*(194) = -3.27, *p* = .01, 95% *CI*: -.34, -.08. Among those with lower satisfaction with life (-1SD), PIQ scores was associated with confidence, *B* = .47, *t*(194) = 4.96, *p* < .001, 95% *CI*: .28, .66. For those with higher satisfaction with life (+1SD), non-significant relationship emerged between the PIQ and confidence *B* = .05, *t*(194) = .38, *p* = .70, 95% CI: -.20, .30. Analyzed differently, this interaction showed that, among participants with low levels of the PIQ (-1SD), satisfaction with life was positively related to confidence, *B* = .50, *t*(194) = 5.07, *p* < .001, 95% *CI*: .30, .70. In contrast, for participants with high levels of the PIQ (+1SD), the positive relation between satisfaction with life and confidence was not significant, *B* = .08, *t*(194) = .83, *p* = .41, 95% CI: -.11, .26.

2.- *Hierarchical regression analysis on the PIQ with confidence, satisfaction with life and the interaction terms as predictor variables (Study 1)*. The main effect of confidence on the PIQ was significant, *B* = .16, *t*(194) = 2.71, *p* = .007, 95% *CI*: .04, 27, indicating that participants scoring higher in confidence reported more scores on the PIQ. The main effect of satisfaction with life on the PIQ was significant, too, *B* = -.25, *t*(194) = -4.50, *p* < .001, 95% *CI*: -.35, -.14, showing that participants with higher punctuations in satisfaction with life reported less PIQ scores. The interaction between confidence and satisfaction with life was significant *B* = -.13, *t*(194) = -2.49, *p* = .01, 95% *CI*: -.23, -.03. Among those with higher confidence (+1SD), satisfaction with life was associated with the PIQ, *B* = -.37, *t*(194) = -5.31, *p* < .001, 95% *CI*: -.51, -.24. For those with lower confidence (-1SD), non-significant relationship emerged between satisfaction with life and PIQ scores *B* = -.12, *t*(194) = -1.47, *p* = .14, 95% CI: -.27, .04. Analyzed differently, this interaction showed that, among participants with low levels on satisfaction with life (-1SD), confidence was positively related to PIQ scores, *B* = .28, *t*(194) = 4.64, *p* < .001, 95% *CI*: .16, .40. In contrast, for participants with high levels on satisfaction with life (+1SD), the relation between confidence and the PIQ was not significant, *B* = .03, *t*(194) = .29, *p* = .77, 95% CI: -.15, .20.

3.- *Hierarchical regression analysis on confidence with the PIQ, satisfaction with life and the interaction terms as predictor variables (Study 2)*. The main effect of the PIQ on confidence was significant, *B* = .43, *t*(54) = 3.32, *p* =.002, 95% CI: .17, 68, indicating that participants scoring higher in the PIQ reported more confidence. Also, the main effect of satisfaction with life on confidence was significant, too, *B* = .48, *t*(54) = 3.81, *p* < .001, 95% CI: .23, 74, showing that participants with higher punctuations in satisfaction with life informed more confidence. The interaction between the PIQ and satisfaction with life was marginally significant *B* = -.19, *t*(54) = -1.81, *p* = .08, 95% CI: -.41, -.02. Among those with lower satisfaction with life (-1SD), PIQ scores was associated with confidence, *B* = .68, *t*(54) = 4.24, *p* < .001, 95% CI: .36, 1.00. For those with higher satisfaction with life (+1SD), non-significant relationship emerged between the PIQ and confidence *B* = .29, *t*(54) = 1.68, *p* = .10, 95% CI: -.06, .63. Analyzed differently, this interaction showed that, among participants with low levels of the PIQ (-1SD), satisfaction with life was positively related to confidence, *B* = .62, *t*(54) = 4.21, *p* < .001, 95% CI: .32, .91. In contrast, for participants with high levels of the PIQ (+1SD), the positive relation between satisfaction with life and confidence was not significant, *B* = .23, *t*(54) = 1.26, *p* = .21, 95% CI: -.14, .60.

4. *Hierarchical regression analysis on the PIQ with confidence, satisfaction with life and the interaction terms as predictor variables (Study 2)*. The main effect of confidence on the PIQ was significant, *B* = .38, *t*(54) = 3.13, *p* = .001, 95% CI: .14, 62, indicating that participants scoring higher in confidence reported more scores on the PIQ. The main effect of satisfaction with life on the PIQ was significant, too, *B* = -.57, *t*(54) = -5.05, *p* < .001, 95% CI: -.80, -.34, showing that participants with higher punctuations in satisfaction with life reported less PIQ scores. The interaction between confidence and satisfaction with life was not significant *B* = -.11, *t*(56) = -1.15, *p* = .25, 95% *CI*: -.31, .08.

5. *Additional analysis with the aggregated data of Studies 1 and 2*. Aggregating across Studies 1 and 2, we conducted a hierarchical regression analysis on confidence with the PIQ, satisfaction with life, Study, and the interaction terms as predictor variables. The main effect of Study on confidence was significant for the collapsed sample, *B* = .16, *t*(252) = 2.55, *p* = .01, 95% *CI*: .04, 28. As expected, confidence was higher in the clinical sample (Study 2) than in the non-clinical sample (Study 1). This finding is consistent with the idea that individuals with delusions tend to have excessive levels of confidence. The main effect of the PIQ on confidence was also significant, *B* = .22, *t*(252) = 3.51, *p* < .001, 95% *CI*: .10, 34, indicating that participants scoring higher in the PIQ reported more confidence. Finally, the main effect of satisfaction with life on confidence was significant, too, *B* = .30, *t*(252) = 5.01, *p* < .001, 95% *CI*: .18, 42, showing that participants with higher punctuations in satisfaction with life informed more confidence. The interaction between the PIQ and satisfaction with life was significant *B* = -.23, *t*(252) = -4.08, *p* < .01, 95% *CI*: -.35, -.12. Among those with lower satisfaction with life (-1SD), PIQ scores was associated with confidence, *B* = .43, *t*(252) = 6.10, *p* < .001, 95% *CI*: .29, .57. For those with higher satisfaction with life (+1SD), non-significant relationship emerged between the PIQ and confidence *B* = -.02, *t*(252) = -.26, *p* = .79, 95% CI: -.21, .16. Analyzed differently, this interaction showed that, among participants with low levels of the PIQ (-1SD), satisfaction with life was positively related to confidence, *B* = .50, *t*(252) = 5.89, *p* < .001, 95% *CI*: .33, .66. In contrast, for participants with high levels of the PIQ (+1SD), the positive relation between satisfaction with life and confidence was not significant, *B* = .04, *t*(252) = .51, *p* = .61, 95% CI: -.11, .19. No other effects were significant, *ps* > .38.

Aggregating across Studies 1 and 2, we also conducted a multiple regression analysis on the PIQ with Study, confidence, satisfaction with life and the interaction terms as predictor variables. Although the main effect of Study on the PIQ was non-significant for the collapsed sample, *B* = -.01, *t*(256) = -.16, *p* = .88, 95% *CI*: -.14, 12, one can expect PIQ and confidence to be related because extreme confidence is a symptom of persecutory delusions. Therefore, we also conducted a one-way ANOVA on the PIQ (as a dependent measure) entering Study as a factor. As expected, PIQ score was higher in the clinical sample (Study 2) than in the non-clinical sample (Study 1), *F*(1, 258) = 5.04, *p* = .03, *η*­_p_^2^ = .02. This finding reveals that PIQ is capable distinguishing between individuals with different levels of delusions (e.g. delusions vs. sub-clinical delusions; Elahi et al., 2017). The main effect of confidence on the PIQ was also significant, *B* = .21, *t*(252) = 3.30, *p* < .001, 95% *CI*: .08, 33, indicating that participants scoring higher in confidence reported more scores on the PIQ. Finally, the main effect of satisfaction with life on the PIQ was significant, too, *B* = -.30, *t*(252) = -5.08, *p* < .001, 95% *CI*: -.42, -.18, showing that participants with higher punctuations in satisfaction with life reported less PIQ scores. The interaction between Study and satisfaction with life was significant *B* = -.22, *t*(252) = -3.39, *p* < .01, 95% *CI*: -.35, -.09. In Study 2, satisfaction with life was strong associated with PIQ scores, *B* = -.59, *t*(252) = -4.44, *p* < .001, 95% *CI*: -.85, -.33. In Study 1 a weaker but significant relationship emerged between satisfaction with life and the PIQ *B* = -.16, *t*(252) = -2.34, *p* = .02, 95% CI: -.29, -.03. Analyzed differently, this interaction showed that, among participants with low levels on satisfaction with life (-1SD), Study was related to the PIQ, *B* = .24, *t*(252) = 3.07, *p* < .001, 95% *CI*: .09, .38. In contrast, for participants with high levels of the PIQ (+1SD), the relation between Study and the PIQ was not significant, *B* = -.13, *t*(252) = -1.31, *p* = .19, 95% CI: -.32, .06. Also, the interaction between confidence and satisfaction with life was significant *B* = -.15, *t*(252) = -2.75, *p* < .01, 95% *CI*: -.26, -.04. Among those with higher confidence (+1SD), satisfaction with life was associated with the PIQ, *B* = -.47, *t*(252) = -6.12, *p* < .001, 95% *CI*: -.62, -.32. For those with lower confidence (-1SD), non-significant relationship emerged between satisfaction with life and PIQ scores *B* = -.11, *t*(252) = -1.31, *p* = .19, 95% CI: --.28, .05. Analyzed differently, this interaction showed that, among participants with low levels on satisfaction with life (-1SD), confidence was positively related to PIQ scores, *B* = .37, *t*(252) = 5.51, *p* < .001, 95% *CI*: .24, .50. In contrast, for participants with high levels on satisfaction with life (+1SD), the relation between confidence and the PIQ was not significant, *B* = .01, *t*(252) = .15, *p* = .88, 95% CI: -.18, .20. No other effects were significant, *ps* > .25.

Finally, aggregating across Studies 1 and 2, we conducted a multiple regression analysis on satisfaction with life with Study, the PIQ, confidence, and the interaction terms as predictor variables. As expected, results of the multiple regression analysis showed the same pattern of Studies 1 and 2, and the key effects remained significant. A significant main effect of the PIQ on satisfaction with life emerged for the collapsed sample, *B* = -.25, *t*(252) = -4.12, *p* < .001, 95% CI: -.37, -.13, and the main effect of confidence on satisfaction with life was also significant, *B* = .22, *t*(252) = 3.69, *p* < .001, 95% *CI:* .10, .34. Furthermore, as expected, the main effect of Study on satisfaction with life was also significant, *B* = -.16, *t*(252) = 2.68, *p* < .01, 95% CI: -.28, -.04, indicating that participants in Study 1 (non-clinical populations) informed more satisfaction with life than participants in Study 2 (clinical population). The predicted interaction between the PIQ and confidence was significant, *B* = -.21, *t*(252) = -3.52, *p* < .001, 95% CI: -0.32, -0.09. For participants scoring high in confidence (+1SD), the PIQ was inversely associated with satisfaction with life, *B* = -.48, *t*(252) = -6.48, *p* < .001, 95% CI: -0.63, -0.34. For those scoring low in confidence (-1SD), the relationship between the PIQ and satisfaction with life was not significant, *B* = -.06, *t*(252) = -.70, *p* = .48, 95% CI: -.24, .11. No other effects were significant, *ps* > .28.

Also, aggregating across Studies 1 and 2, we conducted an ANOVA on satisfaction with life with Study as a factor. As expected, satisfaction with life was higher in the non-clinical sample (Study 1) than in the clinical sample (Study 2), *F*(1, 258) = 7.81, *p* < .01, , *η­_p_*^2^ = .03.
